# Supplementary material for: Are carbon emissions trading and green financial instruments synergistic? -Comprehensive quantitative research based on content analysis
Source: PLoS One. 2024 Mar 7;19(3):e0298601. doi: 10.1371/journal.pone.0298601 (PMC10919601; doi:10.1371/journal.pone.0298601)
Supplement: S1 Data — (ZIP) [file pone.0298601.s001.zip › Data/地级市Z_F工作报告.zip!/╔╜╢1⁄2/╟α╡║╩╨/└┤╫╘╣1⁄2╓┌║┼í╛┬φ┐╦╩2╛▌═°í┐_2010.docx]

各位代表：

　　现在，我代表市人民政府向大会作工作报告,请予审议，并请各位政协委员和其他列席人员提出意见。

　　一、2009年工作回顾

　　去年年初，受国际金融危机的严重冲击，我市经济发展延续上年四季度下行态势，进入了新世纪以来最为困难的一年。一年来，在中共青岛市委的坚强领导下，我们紧紧依靠全市人民，深入贯彻科学发展观，认真落实胡锦涛总书记视察山东及青岛重要讲话精神，坚决执行国家应对国际金融危机的各项政策措施，积极作为，科学务实，全力以赴保增长、保民生、保稳定，一批关系长远发展的项目开工建设，一批惠及城乡民生的实事相继完成，“环湾保护、拥湾发展”战略呈现快速推进、多点突破的良好局面，在建设富强文明和谐的现代化国际城市道路上迈出了新步伐。

　　初步统计，全市生产总值达到4890.3亿元，比上年增长12.2%；地方财政一般预算收入377亿元，增长10.1%；规模以上固定资产投资2458.9亿元, 增长25.3%；社会消费品零售总额1744亿元, 增长19.1%；城市居民人均可支配收入22368元，农民人均纯收入9249元，分别增长9.3%、8.7%；居民消费价格上涨0.5%；城镇登记失业率2.81%；人口自然增长率1.52‰；万元生产总值能耗和主要污染物排放总量持续下降。市十四届人大二次会议确定的目标任务全面完成。

　　（一）坚持把保持经济平稳较快发展作为首要任务，努力扩大投资消费需求，实现经济运行回升向好

　　着力增强投资拉动作用。实施重点项目335个，总投资3374亿元。城市地铁一期奠基开工，董家口港区开发全面实施，青荣城际铁路、海青铁路等获国家批准。民生项目、高新技术和服务业领域投资分别增长45.9%、20.1%和34.2%。争取中央扩内需资金7.3亿元，438个项目全部开工。发行地方债券11亿元。出台促进民间投资意见，民间投资完成1660亿元，增长15.6%。在外需严重萎缩的情况下，投资拉动经济增长10.5个百分点，不仅有力地促进了经济企稳回升，而且为长远发展奠定了基础。

　　着力提高消费贡献率。全面落实国家促进消费各项政策，家电下乡销售额增长30%，汽车类零售额增长34.9%，商品房销售额增长80.3%。万达广场等12个大型商贸项目建成开业。加大旅游促销力度，接待国内外游客和旅游总收入分别增长15.4%、16.4%。成功举办国际海运年会、中国品牌节等177个节庆展会。

　　着力缓解企业生产经营困难。年初，受国际金融危机冲击，全市45%的规模以上工业企业利润下降或亏损。我们迅速采取暂停、取消和降低收费等措施，减轻企业负担。通过政策引导、银企对接，帮助企业争取550亿元授信额度，为中小企业融资190亿元、盘活流动资金140亿元。通过举办青岛名优产品推介会和国际经贸洽谈会，帮助企业开拓市场。通过建立领导干部服务企业制度，强化大企业直通车和中小企业分类服务，保障企业生产经营。全年规模以上工业增加值和利润分别增长14.9%、77.4%。

　　（二）坚持把调整经济结构作为关键环节，在调整中发展、在创新中转型，努力构建科学发展新模式

　　加快工业调整振兴。制定出台了七大产业三年推进方案和新兴产业发展指导意见。投资1100亿元的317个工业振兴项目竣工100个。上汽通用五菱发动机、北船重工海西湾造修船基地等集群项目竣工投产。七大产业产值增长15.3%，占规模以上工业比重达到84.5%。

　　大力发展现代服务业。新引进18家金融机构。新增贷款首次突破1000亿元，达到1205亿元，是上年的1.8倍。华强文化科技产业基地等项目开工，国际动漫游戏产业园等创意园区建成，中远仓储中心等物流项目竣工营业。港口吞吐量和集装箱吞吐量分别达到3.2亿吨、1028万标箱。航空旅客吞吐量达到966万人次，增长17.8%。服务业占生产总值比重达到45.2%，提高1.1个百分点。

　　推动创新资源集聚和关键技术突破。全面加强与中科院战略合作,引进中科院兰州化学物理所等3家科研机构。引进海外留学人才超过1000名，增长50%。海尔集团入选“国家海外高层次人才创新创业基地”。哈尔滨工程大学青岛产学研基地开工。时速350公里动车组等20多项产业关键技术实现突破。获国家科技奖励8项，其中技术发明一等奖1项，科技进步一等奖1项。发明专利授权量增长36.6%，成为“国家知识产权工作示范城市”。制定蓝色经济区建设发展规划纲要和促进高端产业发展意见，成功举办首届蓝色经济发展国际高峰论坛。

　　加强生态文明建设。全面推进建筑节能、绿色照明等十大节能工程，完成140万平方米建筑节能改造，开工可再生能源建筑应用项目120万平方米。完成50个减排项目。实施了崂山水库上游污水治理和过城河道截污工程。新建排水管网265公里，投资额超过前十年总和。市区新增集中供热面积705万平方米，增长41%。完成第二条入市天然气管线工程，垃圾沼气发电项目投入运行。市区空气质量优良率达到91.2%。

　　（三）坚持把“环湾保护、拥湾发展”作为主导战略，大力推进科学规划、集约建设、精细管理, 城市承载力进一步提升

　　加强城市规划管理。拥湾发展专项规划、高新区总体规划、五市新一轮总体规划修编完成。董家口港区总体规划获国家批准。数字化城管指挥中心投入运行。铁路沿线环境综合整治初见成效。通过全国绿化模范城市检查和国家园林城市复查。获得2014年世界园艺博览会承办权。

　　加快重点区域建设。高新区胶州湾北部园区基础设施建设进展迅速，防潮坝工程正式开工，科技大道、创业大道等加快建设，创业中心、企业加速器等主体封顶，一批高新技术产业项目落户。前湾港区新增6个万吨级集装箱泊位。前湾保税港区一期封关运营。市北小港湾、胶州少海新城等节点建设加快推进，海底隧道、海湾大桥、环湾大道等工程进展顺利。老城区企业搬迁改造启动17户，累计实施37户，竣工投产10户。环湾重点区域和重要节点建设，成为全市投资和经济发展的新增长点。

　　（四）坚持把解决好“三农”问题作为重中之重，扎实推进新农村建设，促进城乡统筹协调发展

　　县域经济规模进一步壮大。粮食总产、夏粮单产均创历史最好水平。建成百万亩蔬菜示范区。6个新增工业功能区加快建设。县域产值过10亿元特色产业基地达到23个。五市生产总值和财政收入平均增幅分别比全市高1.3个、2.3个百分点。

　　农村生产生活条件进一步改善。市级财政投入“三农”资金24.9亿元，增长13.9%。63%的行政村达到“五化”标准。43座水库除险加固、9条中小河道整治以及墨水河综合治理等工程完成。实施村庄饮水安全工程，解决了31万人的饮水问题。完成8座大中型沼气建设，新增户用沼气1.1万户。改造100所农村薄弱中小学和13.7万平方米危房，全部免除农村义务教育阶段公办学校寄宿学生住宿费。新型农村合作医疗住院补偿比例提高到43%。农村住房开工建设7.9万户，竣工7.2万户，改造危房8600户。

　　（五）坚持把改革开放作为根本动力，更加注重体制机制创新，发展活力不断增强

　　积极推进重点领域改革。机械总公司与中国节能投资公司战略合作实现突破。组建市交通开发投资中心，理顺了公路管理体制。投资体制改革在提高投融资能力、盘活不良资产、防范财务风险、推进重点项目等方面发挥了重要作用。按照中央和省的部署，政府机构改革稳步实施，医药卫生体制改革有序展开，文化广电新闻出版改革基本完成，文艺院团改革全面启动。组建中小企业公共服务平台，民营经济增加值增长13.9%。

　　去年以来，面对外需急剧下滑的严峻形势，及时出台了促进外贸平稳发展的48项措施，充分发挥外贸企业联席会议作用，及时协调解决突出问题，完成出口退税150亿元，实现出口269亿美元。加大定向招商力度，香港、日韩招商签约项目127个，合同外资76亿美元。全市实际到账外资18.6亿美元，高于预期目标。引进内资465亿元，增长14%。对外经济合作营业额增长37.3％。与韩国蔚山广域市、德国雷根斯堡市结成友好合作关系城市。

　　（六）坚持把保障和改善民生作为最大责任，努力办好顺民意、解民忧、惠民生的实事，民生保障水平持续提高

　　各位代表！去年，在困难多、财力紧的情况下，我们坚持财力惠民不减，保障力度明显加大。市财政用于民生和社会事业的支出87.6亿元，增长26.2%，高于市级地方财政一般预算收入增幅18.1个百分点。为城乡居民办的9件实事全部完成。

　　加强就业和社会保障工作。新增城乡就业38.9万人。扶持创业2.5万人，带动就业7万人。设立2500万元高校毕业生创业资金，近7万名高校毕业生一次性就业率达到88%。企业退休人员养老金和市区、五市失业人员保险金分别提高10%以上，城乡低保救助、农村五保供养和优抚对象抚恤补助标准分别提高15%以上。新增养老床位1700张，改扩建农村敬老院39个。

　　保障性住房和“两改”项目建设效果显著。市区两级投入住房保障资金8.9亿元，年初安排的8000套保障性住房和12个“两改”项目全部开工。2007年以来，开工建设的50个“两改”项目，回迁项目25个、居民2.4万户。3.5万户低收入住房困难家庭实物配租配售比例达到68%。在市区竣工住房总量中，保障性住房和限价商品住房比例达到48%，住房供给结构明显改变。去年，我市房价涨幅低于全国70个大中城市平均水平4.2个百分点，“两改”和保障性住房建设有力地抑制了市场房价过快上涨。

　　社会事业协调发展。坚持优先发展教育。向家庭经济困难学生发放奖学金、助学金2亿元。完成46所市区中小学取暖设施改造，实施了1126所中小学饮水设施更新工程。建成青岛第一国际学校。青岛职业技术学院成为“国家示范高等职业学院”。加快城乡医疗卫生服务体系建设。国医堂、妇女儿童医院迁建、中心医院扩建、西海岸医疗中心等项目主体封顶。甲型H1N1流感和手足口病疫情得到有效控制。繁荣发展文化体育事业。电影《寻找微尘》等荣获全国“五个一工程”奖。全国小提琴比赛、中国国际儿童电影节成功举办。奥帆博物馆、市体育中心一期投入使用，市全民健身中心、老年体育活动中心建成开放。圆满承办“十一运”青岛赛区13项比赛，我市运动员获得全运金牌24.5枚、奖牌60枚，取得历史最好成绩。国家羽毛球队训练基地落户青岛，成功组织了沃尔沃等大型帆船赛事青岛站活动。全面完成北川县曲山镇和陈家坝乡灾后援建工作。

　　（七）坚持把创建全国文明城市作为重要抓手，不断强化社会管理，努力保持社会和谐稳定

　　新一轮文明城市创建取得阶段性成果。涌现出一批全国、全省道德模范和文明单位、文明村镇，在全国公共文明指数测评中取得优异成绩。认真做好宗教工作，切实维护民族团结。做好人口与计划生育工作，低生育水平保持稳定。“双拥”共建活动深入开展，国防教育和国防后备力量建设不断加强，军政军民关系更加巩固，多国海军活动圆满成功。

　　“平安青岛”创建深入开展。积极构建打、防、控相结合的立体化安保格局，群众社会治安满意率进一步提高。扎实推进领导大接访、带案下访工作，信访积案和矛盾纠纷得到有效化解。出台了加快城乡和谐社区建设的意见。大力开展“安全生产责任落实年”活动，全面推行网格化监管，事故总起数和死亡人数分别下降13.5%、17.5%。加强食品药品安全整顿和示范创建工作。健全公共安全应急体系，应急保障能力不断加强。

　　（八）坚持把建设服务型政府作为重要目标，深入开展学习实践科学发展观活动，行政效能和服务水平进一步提高

　　加强民主法制建设。自觉接受市人大及其常委会的法律监督、工作监督和市政协的民主监督，人大代表、政协委员的建议、提案全部办复并实现网上公开，加强了与各民主党派、工商联、无党派人士的对口联系。制定推进依法行政的实施意见，向市人大常委会提请审议7件地方性法规草案。行政审批服务大厅投入使用，有审批职能的政府部门全部进驻，审批效率平均提速65%。加大行政审批清理力度，市级行政许可事项削减调整70%，非行政许可事项削减调整66%。积极推进政务公开，组织政府在线访谈73次、新闻发布90次，分别增加68%、23%。开展政府部门“向市民报告、听市民意见、请市民评议”活动，初步建立了公众评价政府工作的新机制。

　　完善惩治和预防腐败体系。建成公共资源交易大厅和综合电子监察平台。认真抓好治理 “小金库”、商业贿赂和纠风工作。高度重视政府投资项目资金管理和债务管理，建立了偿债责任制和债务风险预警机制。加强了对政府部门执行财经法规、主要领导经济责任的审计监督。

　　各位代表！过去的一年，是青岛在逆势中前进、在转型中发展的一年，也是战胜各种挑战、实现重大突破的一年。一年来，胡锦涛总书记、温家宝总理先后来青视察，为我们攻坚克难增强了信心力量；国家扩大内需政策的部署推进，为我们应对危机创造了有利条件；“环湾保护、拥湾发展”战略的全面实施，为我们抢抓机遇拓展了新的空间。一年来，面对特殊时期、特殊困难，全市人民、社会各界和衷共济，迎难而上，为实现经济社会平稳较快发展作出了突出贡献。在此，我代表市人民政府，向全体市民和外地来青建设者，向各民主党派、工商联、无党派人士、各人民团体，向中央、省驻青单位和人民解放军驻青部队、武警官兵、公安干警，向所有关心、支持青岛建设发展的海内外朋友，表示衷心的感谢和崇高的敬意！

　　我们也清醒地看到，当前经济社会发展还存在不少问题：经济回升的基础还不牢固，社会投资意愿不高，居民消费后劲不足，经济增长内生动力不强；产业结构层次偏低，体制机制创新步伐不快，各类人才特别是高层次人才严重不足，转变发展方式和改革攻坚任务十分艰巨；城乡发展不平衡依然突出，城市管理还不适应快速发展的需要；城乡居民收入持续增长和社会保障可持续发展难度加大，老年服务设施建设滞后。政府工作与人民期待相比存在较大差距：对发展机遇期和矛盾凸显期的认识把握有待加强，社会管理和公共服务能力有待提高；一些部门协调配合不够，形式主义、官僚主义现象仍然不同程度地存在，极少数人甚至违法违纪、以权谋私、贪污腐败。对此，我们将进一步解放思想，拓宽视野，采取有针对性的措施，努力加以解决。

　　二、2010年工作总体要求

　　2010年，是实施“十一五”规划的最后一年，也是应对国际金融危机的关键之年。政府工作的总体要求是：以邓小平理论和“三个代表”重要思想为指导，深入贯彻科学发展观，全面落实党的十七大和十七届三中、四中全会精神，按照中央、省经济工作会议和市委十届八次全委会的部署，全面实施“环湾保护、拥湾发展”战略，加快建设蓝色经济区和高端产业聚集区，着力促进经济发展方式转变和经济结构调整，着力推进改革开放和自主创新，着力改善民生质量和维护社会稳定，保持经济社会平稳较快发展，全面完成“十一五”规划目标任务。

　　经济社会发展的主要预期指标是：全市生产总值增长11%；地方财政一般预算收入增长10%左右；城市居民人均可支配收入和农民人均纯收入分别增长8%；城镇登记失业率控制在4%以内；居民消费价格涨幅控制在3%左右。主要约束指标是：万元生产总值能耗下降4%，二氧化硫和化学需氧量排放量分别控制在11.45万吨和4.75万吨以内；人口自然增长率控制在4‰以内。

　　在工作中，重点把握好四个方面：

　　——加快经济发展方式转变。把转方式、调结构作为主攻方向，着力引进国内外大企业、带入高端要素，着力引进大型科研院所、带入高端人才，进一步优化发展空间和发展环境；坚持自主创新与培育战略性新兴产业相结合，加快产业结构调整步伐，加快县域工业化、城镇化进程，努力提高经济增长的质量和效益。

　　——全面推进改革开放。把深化改革与促进发展结合起来，把扩大内需与稳定外需结合起来，加快制度创新，提升开放水平，消除影响经济社会发展的体制机制障碍，增强经济发展的内生动力和竞争力。

　　——切实保障和改善民生。把改善民生、发展社会事业作为扩内需、调结构的重点，更加注重解决人民群众切身利益问题，促进公共资源配置更多地向民生倾斜，推动基本公共服务均等化，保持社会安定祥和，让城乡居民分享更多的发展成果。

　　——加强服务型政府建设。把公共服务和社会管理放在更加突出的位置，坚持依法行政，转变政府职能，自觉接受社会监督，加快创建政府效能最高、行政成本最低城市，更好地为发展服务、为基层服务、为群众服务。

　　各位代表！今年我们面临的经济形势依然十分复杂，不确定、不稳定和不可预料的因素较多。我们必须准确判断形势，进一步增强忧患意识和风险意识，更加周密地超前应对，更加科学地谋划发展，更加有效地破解难题，努力在新一轮调整转型中抢抓机遇，在科学发展中增创优势，为建设经济文化强省作出新贡献！

　　三、加快推进经济发展方式转变

　　按照“痛下决心、狠下功夫、打好硬仗”的要求，转变观念，调整思路，力求在优化需求结构、促进产业升级上实现更大突破，在加快自主创新、建设生态文明上取得实质性进展。

　　（一）积极有效地扩大消费和投资需求

　　进一步增强消费拉动力。继续落实好国家扩大消费各项政策，不断改善消费环境。抓好商贸集聚区、商业特色街和大型专业批发市场规划建设，大力发展品牌连锁经营。扩大文化娱乐、休闲健身、信息通信等服务消费。出台进一步促进旅游业发展的政策措施，推进港中旅海泉湾等旅游项目和旅游公共服务平台建设，把旅游业培育成战略性支柱产业。支持居民自住和改善性购房需求，抑制投资投机性购房，规范房地产市场秩序，促进住房消费健康发展。

　　努力保持投资稳定增长。今年安排重点项目240个、总投资2828亿元。年内实现海底隧道和海湾大桥主线贯通，确保2011年建成通车。加快建设城市地铁一期和铁路客运北站，开工建设新疆路高架及重庆路快速路，完成环湾大道、东西快速路三期工程。协调推进蓝烟铁路电气化改造、青连铁路、青龙高速公路、宁东送电、中石油海洋工程等项目。积极发展多种投融资模式，完善鼓励民间投资的相关政策，不断扩大社会投资，切实保护投资者的合法权益。

　　（二）加快构建富有竞争力的现代产业体系

　　提高现代服务业发展水平。深化金融聚集区建设，加快发展银行、保险、证券、信托等法人金融机构。扩大私人银行业务，建设区域性财富管理中心。推进胶州湾国际物流园和临空经济物流项目建设，吸引国际航运、航空公司增辟新航线。推动物联网和互联网结合，推广传感网应用，抓好市民“一卡通”和亚太新国际海底光缆登陆工程。引进国内外知名展会和展览公司，进一步打造品牌展会。积极培育演艺、出版和动漫创意产业，加快建设国家软件出口创新基地、动漫创意产业基地、服务外包基地。大力发展总部经济。鼓励鉴证、科技等领域知名中介服务机构来青落户，支持本地中介服务机构做大做强。

　　促进工业调整振兴。大力发展新能源、新材料、生物医药、节能环保等战略性新兴产业，建成国家生物产业基地医药中试中心，推进北电能源、大唐风电场等新能源项目建设。不断提高主导产业集群配套能力，力争高速动车组产业化基地等100个项目竣工，中石化LNG等项目开工。促进工业化与信息化融合发展，加快纺织服装等传统产业升级改造，推动数字化橡胶轮胎等高新技术产业基地建设。进一步完善大企业直通车、企业联络员等制度，做好煤电油气运保障工作，及时帮助企业解决生产经营难题。

　　（三）着力提高自主创新能力

　　大力实施科技创新工程。积极引进大型科研院所，加快中科院系统在青研发机构建设，筹建高新区国际科技合作园，组建工业技术研究院。深化产学研合作，引导企业与科研院所组建产业技术创新战略联盟，积极承接国家重大科技项目。实施200个过千万元工业技术改造项目，完成1000项技术创新重点项目，组织实施电子信息、轨道交通、生物医药等重点领域关键技术攻关。

　　以自主创新提升品牌竞争力。实施标准化战略，完善政府引导、企业主体、社会补充的技术标准投入机制，形成一批拥有自主知识产权的专利技术和标准。实施全面质量管理战略，加快传统品牌转型升级，重点培育一批装备制造业品牌和高新技术品牌。抓好产品、工程和服务领域质量工作，建设出口产品质量安全示范区。

　　（四）积极推进人才强市战略

　　加大高层次人才引进力度。实施人才投资优先，把人才作为经济社会发展的第一资源摆在突出位置。围绕产业结构调整和文化、教育、卫生、体育等社会事业发展需求，深入实施“300引进海外高层次人才计划”和“222引才工程”，大力引进国内一流、有影响的学科带头人。规划建设一批市级高层次人才创新创业基地。加强“金蓝领培训”，完善专业技术人员继续教育机制，加快培养高技能人才。

　　优化人才发展环境。完善促进优秀人才创新创业的政策措施，健全国际人才交流平台，加强留学人员创业园、博士后流动站等载体建设。创新人才评价选拔和奖励激励机制，进一步营造尊重劳动、尊重知识、尊重人才、尊重创造的浓厚氛围。

　　（五）大力发展低碳经济

　　着手编制低碳经济发展规划，加快建立以低碳排放为特征的产业体系和消费模式，努力建设低碳城市。加大低碳经济、循环经济宣传，树立绿色发展理念和绿色城市形象。积极开展低碳经济试点，高起点规划建设低碳产业园区，开发与国际标准接轨的产品和技术。继续推进十大节能工程和重点行业节能，加大既有建筑节能改造力度，加强对110家重点用能单位目标责任考核和100家重点排污企业环境监管，确保完成“十一五”节能减排目标。

　　实施“十绿”工程，增加森林碳汇。从今年起，每年造林13万亩，五年内全市森林覆盖面积超过500万亩。启动太平山中央公园改造工程，推进浮山保护性建设管理。举全市之力，认真做好2014年世界园艺博览会筹备工作。

　　四、加快推进重点区域建设发展

　　把实施“环湾保护、拥湾发展”战略与建设蓝色经济区、高端产业聚集区相结合，更加注重环湾保护，加快推进拥湾发展，积极推动七区一体、城乡统筹协调发展。

　　（一）加快蓝色经济区和高端产业聚集区建设

　　围绕建设蓝色经济区，编制完成总体发展规划和专项规划。着眼于科学开发利用海洋资源，进一步理清海洋优势产业发展重点，规划建设一批重大项目、示范园区和产业基地。围绕建设高端产业聚集区，努力打造先进装备制造业基地、高技术产业基地和现代服务业基地。制定蓝色经济区和高端产业聚集区扶持政策，建立相关产业统计指标与核算体系。

　　（二）加强环湾保护和重点区域开发

　　做好环湾保护与发展规划。实施《青岛市海洋环境保护规定》，切实加强胶州湾及近海海域生态保护和污染防治，推进海洋、海岛、湿地生态保护区规划建设，进一步完善监管措施，提高环境监测能力。加强环湾区域交通、公用事业等专业规划修编和地铁沿线规划控制。完成城市总体规划报审工作，加快土地利用总体规划修编，完善市域城镇规划体系。

　　加快重点园区发展。对高新区继续实施饱和性投资，完成防潮坝等基础设施工程，开工大学科技园主园区，同步推进定向招商、项目建设、机制创新。推动青岛经济技术开发区省级综合配套改革试点，培育新的增长点。加快董家口港区开发，做好一类口岸开放准备工作。抓好前湾保税港区运营和二期基础设施建设。

　　推动环湾节点建设实现新突破。加快市南国际航运服务区、四方欢乐滨海城、李沧交通商务区、城阳出口加工区等节点开发，加强红岛区域规划和基础设施配套。完善老城区企业搬迁改造政策，新启动15户企业搬迁。

　　（三）大力推进县域工业化和城镇化

　　提高县域经济整体水平。增强国家级、省级工业园区和6个工业功能区的承载能力，积极承接市区工业企业转移，吸引跨国公司、大企业和知名品牌落户，培育一批带动作用强的规模以上工业企业。促进县域服务业与工业协调发展，重点发展与工业园区配套的物流、科技与信息等生产性服务业。加快建立金融、科技、人才等支撑县域经济发展的保障体系。

　　加快推进新型城镇化。实施以道路交通为重点的城乡一体化基础设施建设，优化城镇空间布局，启动一批城镇组团、重点中心镇和农村新型社区建设。深化户籍制度改革，增强城镇集聚效应。做好第三批国家级小城镇发展改革试点。新建改建农村公路500公里，完成中型病险水库除险加固和村庄“五化”工程。实施城乡供水一体化改造，解决143个村庄集中供水问题。扶持建设7000个户用沼气。以改善地貌、林貌、河貌、村貌为重点，实施路域综合治理。新建农村住房4.7万户，改造农村危房8100户。

　　做大做强现代农业。全面落实各项强农惠农政策，扩大农业政策性保险试点范围。切实抓好粮食生产，加快建设百万亩花生、果茶示范区。培育一批现代农业园区和标准化规模养殖基地，改造提升传统水产养殖业。积极推进以良种繁育为重点的农业科技创新，完善基层农技推广服务体系。深化农村“三大合作”，扶持发展农产品加工龙头企业和“四化”合作社，加快农业产业化步伐。

　　五、全面深化改革扩大开放

　　着眼于解决经济社会发展中的深层次矛盾，加大体制机制创新力度，加快转变对外经济发展方式。

　　（一）深化重点领域和关键环节改革

　　推进国有企业开放性、市场化改造重组，鼓励国有企业与国内外大企业战略合作，推动产权多元化。深化国有资产经营公司改革，完善国有资本经营预算、风险防范制度。加快投资体制改革，提高国有投资公司投融资功能和防范债务风险能力。支持企业在境内外资本市场上市融资。进一步加强政府性债务管理，有效防范财政风险。深化医药卫生体制改革，加快建立覆盖城乡居民的基本医疗卫生制度，稳妥抓好公立医院改革试点。有序推进农村土地管理制度改革，提高集约节约用地水平。做好集体林权制度改革，明晰产权，放活经营权。稳步推进政府机构改革，建立完善职责明确、权责清晰的行政责任体系。深化文化体制改革，推进国有文艺演出院团改制工作。加快供销合作社改革发展。

　　（二）切实增强小企业发展活力

　　着力改善小企业、微型企业的发展环境。健全公共服务平台，积极提供信息咨询、技能技术培训、营销网络和信贷融资、法律援助服务。落实对小型微利企业的税收优惠政策。引导银行与担保机构合作，积极发展村镇银行和小额贷款公司。继续实施小企业成长工程，设立创业投资引导基金，吸引社会风险投资，扶持一批高成长性科技企业。加大政策宣传和典型推广，建立小企业对政府部门服务评议制度，营造良好的发展氛围。

　　（三）促进外经贸稳定发展

　　按照“保份额、调结构、促平衡”的要求，推动出口稳步回升。积极扩大自主知识产权、自主品牌、自主营销产品出口。鼓励企业在巩固传统出口市场的同时，大力开拓新兴市场。完善国际贸易摩擦和贸易壁垒应对机制。扩大出口信用保险覆盖面。

　　全面推进定向招商，切实提高引资质量。重点引进先进制造业、现代服务业、高新技术产业、节能环保产业和新能源项目，抓好香港、日韩招商签约项目的落实，力争工业和服务业定向引进外资分别增长25%和30%以上。支持企业参与境外资源开发，收购境外优质资产、研发机构、知名品牌和营销网络。

　　加强国际国内交流合作。做好外事、侨务、对台工作，办好东亚经济交流推进机构第四届大会。继续做好对西藏、贵州、三峡库区、菏泽、曹县等地的对口支援工作，加强与日照、潍坊、德州等省内城市的交流合作。全面完成援川重建任务。

　　六、加强以改善民生为重点的社会建设

　　坚持以人为本、民生为重，大力实施民生工程和发展社会事业，尽心解难事，诚心办实事，热心做好事，确保完成向人民群众承诺的9件实事。

　　（一）提高城乡居民生活水平

　　稳定和扩大就业。完善城乡一体的公共就业服务、人力资源市场和职业技能培训体系，新增城乡就业20万人。统筹做好高校毕业生、就业困难人员和农民工就业，切实增加城乡居民收入。强化创业公共服务，推进创业孵化基地、示范基地和培训基地建设，扶持自主创业2万人，培训企业职工、失业人员和农村劳动力8万人。

　　做好社会保障工作。以保基本、广覆盖、多层次、可持续为重点，启动城乡居民社会基本养老保险制度，加快推进医疗保险、工伤保险、失业保险市级统筹，做好养老保险关系转移接续工作。继续提高企业退休人员基本养老金，月人均养老金由1386元增加到1561元。城镇职工基本医疗保险最高支付限额提高到15万元。城镇居民医保补助标准达到人均120元，新农合人均筹资标准达到150元。制定农村临时救助制度。完善物价上涨与困难群众生活补贴联动机制。

　　加快保障性住房建设。统筹推进“两改”项目，加快中小户型、中低价位住房建设。新建筹廉租住房3000套、经济适用住房5000套；新启动“两改”项目18个，惠及居民1.5万户；综合整治旧住宅区3片，改善6000户居民的居住条件。引导知名房地产企业参与“两改”连片综合开发，打造精品工程。启用住房保障信息系统，加强保障性住房配租配售管理，依法规范拆迁回迁，确保公开公平公正。做好新一轮住房保障三年规划编制工作。

　　完善社会养老服务体系。鼓励社会力量兴办养老服务机构，促进居家养老、社区养老、机构养老规范化建设，新增养老床位4000张以上。实施农村五保供养办法。加强养老服务队伍建设，推进养老服务行业管理。积极支持各区市规划建设老年活动中心和老年大学，广泛开展适合老年人特点的文化体育活动。做好全国老年友好城市试点工作。

　　（二）促进社会事业繁荣发展

　　提高教育现代化水平。逐步统一县域内义务教育学校生均公用经费基本标准。推进以抗震除险为重点的中小学校舍安全工程。完成农村150所薄弱中小学改造，加快新建小区配套学校建设。深化中小学课程改革和招生考试制度改革，推动优质师资合理流动。做好中职学校专业设置和布局调整，落实农村家庭经济困难学生和涉农专业学生免学费政策。加强与国内外知名高校的合作，支持驻青高校引进高层次人才、推进重点学科建设。重视发展幼儿教育、成人教育和特殊教育，促进民办教育健康发展。

　　提高医疗卫生服务质量。结合医药卫生体制改革，完善基层公共卫生和医疗卫生服务体系，推广国家基本药物，严格执行“零差率”销售。启动市第三人民医院、胸科医院迁建工程，确保国医堂等四个大型医院项目全面竣工。积极开展人民满意的医疗卫生机构创建活动，构建和谐的医患关系。扶持中医药事业发展。全力做好重大传染性疾病防治工作。通过国家卫生城市复审。

　　繁荣发展文化事业。以参与筹备第十届中国艺术节为契机，加快重点文化设施改造升级，规划建设青岛文化中心，确保青岛大剧院交付使用。加强基层文化设施建设，推进益民书屋、广播电视村村通等工程。办好全国小提琴考级比赛、青岛音乐节和全民读书月等活动。依法保护历史文化遗产，加强文化市场综合执法。做好第三次全国文物普查工作。

　　大力发展体育事业。广泛开展全民健身活动，完善全民健身服务体系，鼓励各类体育设施向社会开放。做好第16届亚运会和第22届省运会备战参赛工作。积极引进高水平体育赛事，办好国际帆船周和奥帆城市市长论坛等活动。

　　促进各项事业全面发展。增强全民国防观念，积极支持国防建设，推进“双拥”共建，做好人民防空、民兵预备役和复员转业军人安置工作，巩固发展军政军民团结。全面贯彻党的民族宗教政策，促进民族团结进步，依法管理宗教事务。加强人口与计划生育服务管理，稳定低生育水平。组织好第六次全国人口普查。编制实施新一轮妇女儿童发展纲要。发挥红十字会人道救助作用，加强现场救护和避险知识的培训普及。推动慈善事业健康发展。争创全国残疾人工作示范城市。做好档案、史志、气象、防震、仲裁等工作。

　　（三）切实改善居民生活环境

　　抓好水电气暖管网建设，改造排水管网160公里和供水管网65公里，完成10座城镇污水处理厂新建、扩建和升级改造，推进老城区架空电线入地。加快中石油管输天然气配套建设，实现“双气源”供气。做好旧城改造供热站规划建设，进一步增加热源，市区新增集中供热面积810万平方米。加强小涧西垃圾场污染治理。深化公交行业改革，优化公交线路，更新公交车1200辆，市区达到国Ⅲ排放标准的环保车辆超过70%。提高城市道路交通综合管理水平，缓解日益严重的交通压力。加强市政设施日常养护，做好城市集中亮化工作，加大违法建筑、施工扬尘等综合整治力度。

　　（四）努力提升城市文明程度

　　加强社会主义核心价值体系建设，做好新一轮文明城市创建工作。全面实施文明素质提升、市容环境清洁、窗口服务达标、交通秩序畅通、社会文化环境净化“五大行动”，创建一批文明示范标志区域，推出一批文明服务示范窗口。加强社会诚信体系建设。办好未成年人思想道德教育“社会课堂”。深化“新农村、新家园”主题教育活动。充分发挥工会、共青团、妇联等群众组织作用，加强志愿者队伍建设，提高服务水平。繁荣哲学社会科学，推动科学普及工作。

　　（五）营造安定祥和的社会环境

　　切实抓好安全生产。坚持严格的安全生产标准，落实企业主体责任，全面推进网格化、数字化监管。加强安全生产培训教育，深入开展交通、消防、非煤矿山、危险化学品、渔业生产等重点领域安全隐患排查治理，坚决遏制重特大事故发生。健全食品药品安全保障体系，加强食品安全监管综合协调，严厉打击食品药品制假售假行为，全力打造全国食品最安全城市。

　　创新社会管理与服务。鼓励居民参与社区管理，充分发挥社区组织在协调利益、化解矛盾、排忧解难中的作用，引导群众依法反映意愿和诉求，及时帮助解决实际困难。积极培育社会公益性组织，加强社会工作者队伍建设。积极支持各类行业协会、商会参与社会管理和公共服务。完善流动人口管理服务机制。做好律师、公证和法律援助工作。依法组织好第十届村（居）委会换届选举。

　　大力推进平安青岛建设。全面强化社会治安综合治理，进一步健全社会治安防控体系，严厉打击黑恶势力和违法犯罪活动。建立完善社会治安和稳定隐患的治理机制，从源头上预防各类案件和群体性事件发生。开展信访积案清理和重复信访专项整治，及时化解社会不和谐因素。加强公共安全应急队伍建设，完善应急联动机制，不断提高应急处置能力。

　　七、努力建设人民满意的服务型政府

　　各位代表！当前，我市正处在经济社会发展重要战略机遇期和社会矛盾凸显期。社会经济关系的新变化，利益主体的多元化，社会诉求的多样化，要求政府必须坚持依法行政，切实提高公信力和执行力；政府工作人员必须坚持为民、务实、清廉、高效，切实维护社会公平正义。

　　（一）全面提高依法行政和科学决策水平

　　严格按照法定权限和程序行使权力、履行职责、规范行为。自觉接受市人大及其常委会的法律监督和工作监督，主动接受市政协的民主监督。认真执行市人大及其常委会的决议，坚持重大事项、依法行政情况报告制度，提高人大议案、代表建议和政协提案的办理质量。真诚接受民主党派、工商联和无党派人士的监督。坚持重大事项集体讨论决定，完善公众参与、专家论证和政府决策相结合的机制，健全社会听证制度。对关系群众切身利益的重大事项和重点项目，建立风险评估机制。

　　（二）切实增强政府工作透明度

　　以政府投资项目、公共政策、公共服务为重点，扩大政务公开范围，建立财政专项资金预算公开和公众监督机制，保障人民群众的知情权、参与权、表达权和监督权。加快集中配置行政审批职能改革，扩大联合审批和网上受理，建立审批流程动态优化机制。深化电子政务应用，提高社区、村镇政务覆盖面。加强网络和舆情引导，高度重视和及时回应媒体反映的社会热点问题，自觉接受舆论监督。健全政府新闻发布制度，继续办好市长公开电话、政府在线访谈、民生在线和行风在线，完善公众评价机制，促进政府工作在更大范围内接受人民群众的评议和监督。

　　（三）加强作风和廉政建设

　　坚持工作重心下移，深入基层，深入群众，狠抓工作落实。加强学习型组织建设，提高行政效能和工作人员素质，增强推动科学发展、促进社会和谐的能力。完善有利于科学发展的政绩考核评价体系，注重群众公认和实绩，强化行政问责。继续严格控制公用经费和一般性开支，严格控制各类评比达标、表彰庆典活动，坚决制止铺张浪费。

　　加强反腐倡廉建设，积极探索制度与科技相结合的预防腐败新机制，拓展从源头上防治腐败的工作领域。推进电子监察系统向区市和市直部门延伸，逐步实现全程电子监察。严格公共资源交易监督管理。强化对重点领域、重点资金、重点部位的审计监督，依法推进审计公开。严肃查办和惩治各类腐败案件。政府工作人员特别是领导干部，要严格遵守中央和省、市委加强党风廉政建设的各项规定，正确行使人民赋予的权力，严于律己、廉洁奉公，以反腐倡廉的实际行动赢得人民群众的信任。

　　各位代表！编制青岛市国民经济和社会发展第十二个五年规划，是今年的一项重要工作。我们将深入研究事关城市发展的全局性、战略性问题，关注民生，尊重民意，集中民智，编制好“十二五”规划纲要及各专项规划。

　　此同时作用加强范物业管理，，房、医疗、教育和养老等方面的工作，抓好安全生产，创造和谐的社会环境。各位代表！在科学发展观的引领下，青岛已进入转型发展、创新发展、和谐发展的新阶段。让我们更加紧密地团结在以胡锦涛同志为总书记的党中央周围，在中共青岛市委的坚强领导下，开拓进取，扎实工作，全面完成今年和“十一五”规划的目标任务，为建设富强文明和谐的现代化国际城市而努力奋斗！
